# A Method to Evaluate Genome-Wide Methylation in Archival Formalin-Fixed, Paraffin-Embedded Ovarian Epithelial Cells

**DOI:** 10.1371/journal.pone.0104481

**Published:** 2014-08-18

**Authors:** Qiling Li, Min Li, Li Ma, Wenzhi Li, Xuehong Wu, Jendai Richards, Guoxing Fu, Wei Xu, Tameka Bythwood, Xu Li, Jianxin Wang, Qing Song

**Affiliations:** 1 Department of Obstetrics and Gynecology, First Affiliated Hospital, Xi'an Jiaotong University, Xi'an, Shaanxi, China; 2 Cardiovascular Research Institute, Morehouse School of Medicine, Atlanta, Georgia, United States of America; 3 School of Information Science and Engineering, Central South University, Changsha, China; Ohio State University, United States of America

## Abstract

**Background:**

The use of DNA from archival formalin and paraffin embedded (FFPE) tissue for genetic and epigenetic analyses may be problematic, since the DNA is often degraded and only limited amounts may be available. Thus, it is currently not known whether genome-wide methylation can be reliably assessed in DNA from archival FFPE tissue.

**Methodology/Principal Findings:**

Ovarian tissues, which were obtained and formalin-fixed and paraffin-embedded in either 1999 or 2011, were sectioned and stained with hematoxylin-eosin (H&E).Epithelial cells were captured by laser micro dissection, and their DNA subjected to whole genomic bisulfite conversion, whole genomic polymerase chain reaction (PCR) amplification, and purification. Sequencing and software analyses were performed to identify the extent of genomic methylation. We observed that 31.7% of sequence reads from the DNA in the 1999 archival FFPE tissue, and 70.6% of the reads from the 2011 sample, could be matched with the genome. Methylation rates of CpG on the Watson and Crick strands were 32.2% and 45.5%, respectively, in the 1999 sample, and 65.1% and 42.7% in the 2011 sample.

**Conclusions/Significance:**

We have developed an efficient method that allows DNA methylation to be assessed in archival FFPE tissue samples.

## Introduction

Epigenetics is the study of heritable changes in gene expression that are not attributable to alterations in the DNA sequence. DNA methylation is a well-known epigenetic marker that plays an important role in the control of gene activity and the architecture of the nucleus [1]. Evaluation of the level of methylation at all cytosine nucleotides in an individual's genome (called the “methylome”) has recently become possible with the advent of next generation sequencing techniques, specifically sodium bisulfite sequencing [2], [3]. Many different PCR-based methods for the detection of DNA methylation have been developed [4].

Archived human tissues, with known clinical follow-up, represent a valuable resource, particularly for retrospective genetic and epigenetic studies and identification of biological markers that might be useful for risk prediction of disease or prognosis [5]. Biopsied or surgically excised tissues obtained for routine histopathological analysis and diagnosis are widely available, and often formalin and paraffin embedded (FFPE) for decades. However, to date there is no suitable platform to assay genome-wide methylation in these widely available samples. With the increasing interest in understanding the genetic and epigenetic basis of diseases, the ability to extract DNA from these FFPE samples represents an invaluable source of diagnostic material that can be used for genomic analyses and translational studies.

Using DNA from old FFPE tissue may be problematic, as the DNA is often degraded and only limited amounts may be available. The quality of FFPE specimens decreases with time [6] because of linking of nucleic acids and proteins, as well as fragmentation of nucleic acids [5]. Historically, FFPE samples were not considered as a viable source for molecular analyses because the nucleic acids may be heavily modified by protein-nucleic acid and protein-protein cross linking [4]. Furthermore, sodium bisulfite treatment [6], which preserves methylation marks, and as such is necessary for PCR-based studies of DNA methylation, may further degrade the DNA. However, the effect of storage time on the ability to detect genome-wide methylation in FFPE tissues has not yet been documented.

Laser-assisted microdissection is a proven method for isolating specific cell populations for molecular profiling [7]. It can cut particular cells of interest from a tissue section attached to an underlying membrane. In the present study, the identification of ovarian epithelial cells relied on morphological cell characteristics observed after routine histological staining. The system is based on an infrared laser that captures the ovarian epithelial cells of interest from tissue sections mounted on glass slides [7].

In this study, we extracted DNA from epithelial cells within human ovarian FFPE tissues that were obtained from two different individuals and stored for different numbers of years. Using our DNA extraction method, we tested and compared the whole genomic DNA methylation levels in these differentially aged samples using a series of steps including bisulfite conversion, whole genome amplification, purification of amplification, sequencing using a Junior 454 sequencer, and bioinformatics analysis.

## Results

### Overview of ovarian epithelial cell methylation

**Figure 1:**
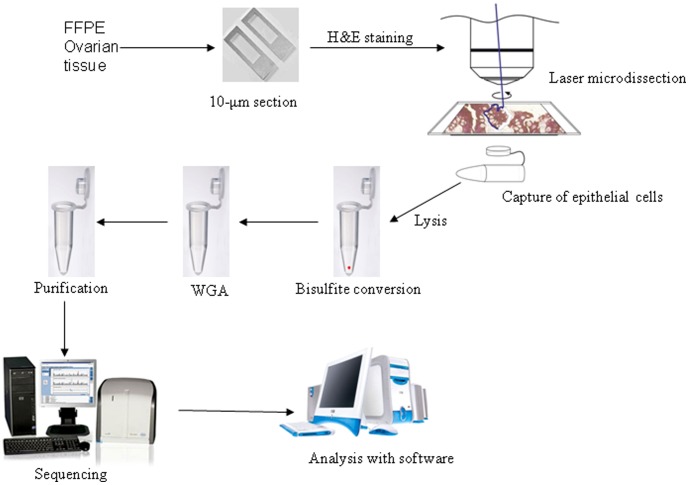
Overview of the procedure for assessing DNA methylation in ovarian epithelial cells. FFPE: Formalin-fixed, paraffin-embedded; H&E staining: Hematoxylin and Eosin staining; WGA: whole genome amplification.

To compare the genome-wide DNA methylation patterns in FFPE ovarian tissue samples stored since 1999 (O1999) or 2011 (O2011), we analyzed their methylation status after bisulfite treatment. The optimized workflow for DNA methylation analysis is summarized in Figure 1, and described in more detail in the Methods. Ten-micron tissue sections were prepared and stained with H&E. Epithelial cells were captured using laser microdissection and put into lysis buffer to obtain genomic DNA. Whole genomic DNA was bisulfite-converted using the EZ DNA Methylation-Direct Kit (Cat. D5021, ZYMO RESEARCH), and then amplified using the EpiTect Whole Bisulfitome Kit (Cat. 59203, QIAGEN). After PCR purification, a Roche 454 sequencer was used to assess the bisulfite sequencing. Associated software performed the alignment and mapping to the original sequence.

### Quantity of purified DNA after whole genomic amplification

Initially, we aimed to verify that DNA methylation measurements could be reliably obtained from FFPE ovarian epithelial tissues. After bisulfite conversion, whole genomic amplification with 50 PCR cycles, and purification, the concentration of DNA was quantified by UV spectrophotometry with a NanoDrop ND-1000 spectral photometer (Nanodrop Technologies; Wilmington, DE). The average concentration of DNA obtained by this method from ovarian epithelial cells from FFPE samples was (3862.8±783.3) ng. This yield indicated that DNA loss was not as high as previously anticipated during sodium bisulfite conversion. Nonetheless, this quantity of DNA was less than that obtained from cells from non-FFPE samples, which in our research group was typically 5–8 µg DNA from each preparation [11]. In repeated measurements, we observed a high degree of consistency in the amount of DNA obtained from the same tissue preparation, indicating that converted DNA could be reproducibly obtained from epithelial cells in FFPE ovarian tissue specimens.

### Processing and analysis of sequence reads

**Figure 2:**
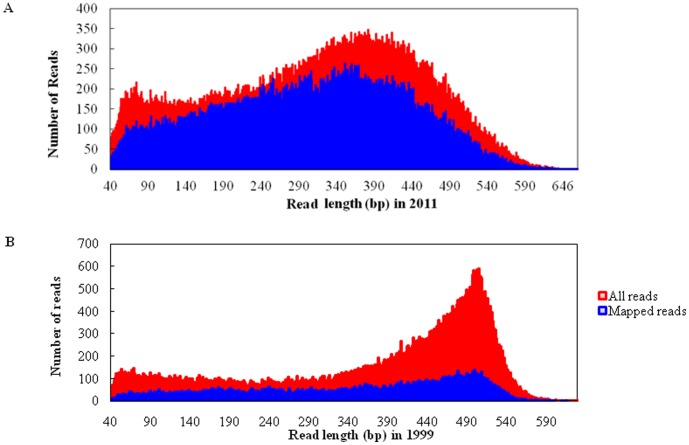
Length distribution of mapped reads compared with all reads. A. Histogram for sample O2011. B. Histogram for sample O1999.

**Figure 3:**
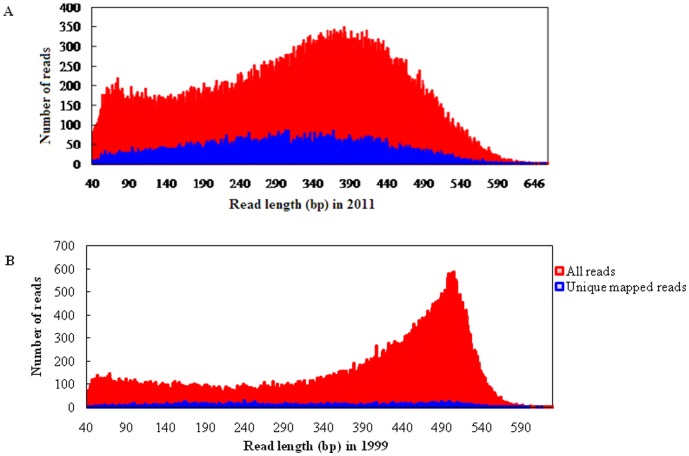
Length distribution of unique mapped reads compared with all reads. A. Histogram for sample O2011. B. Histogram for sample O1999.

**Figure 4:**
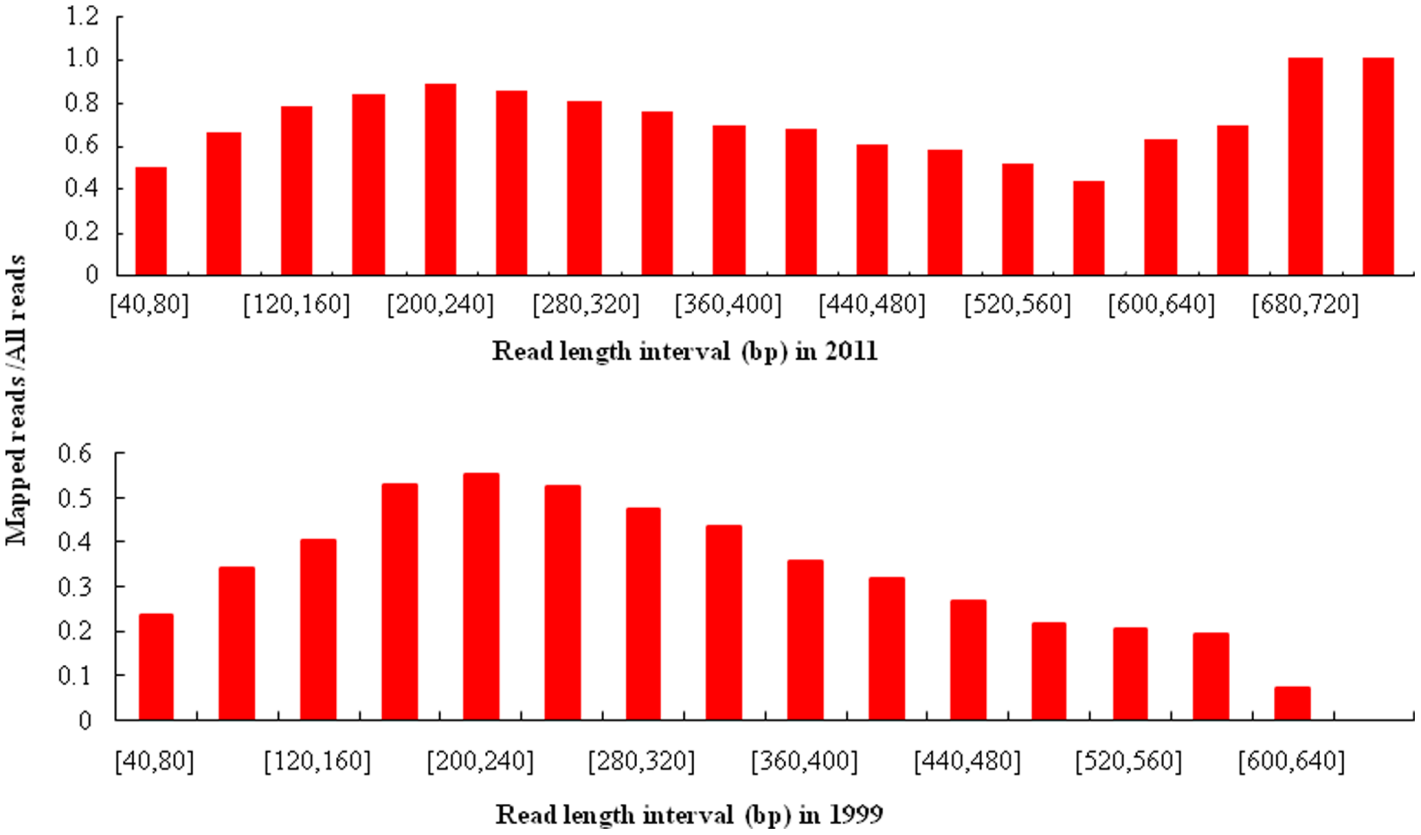
Ratio of mapped reads to all reads according to read length interval. A. Histogram for sample O2011. B. Histogram for sample O1999.

**Figure 5:**
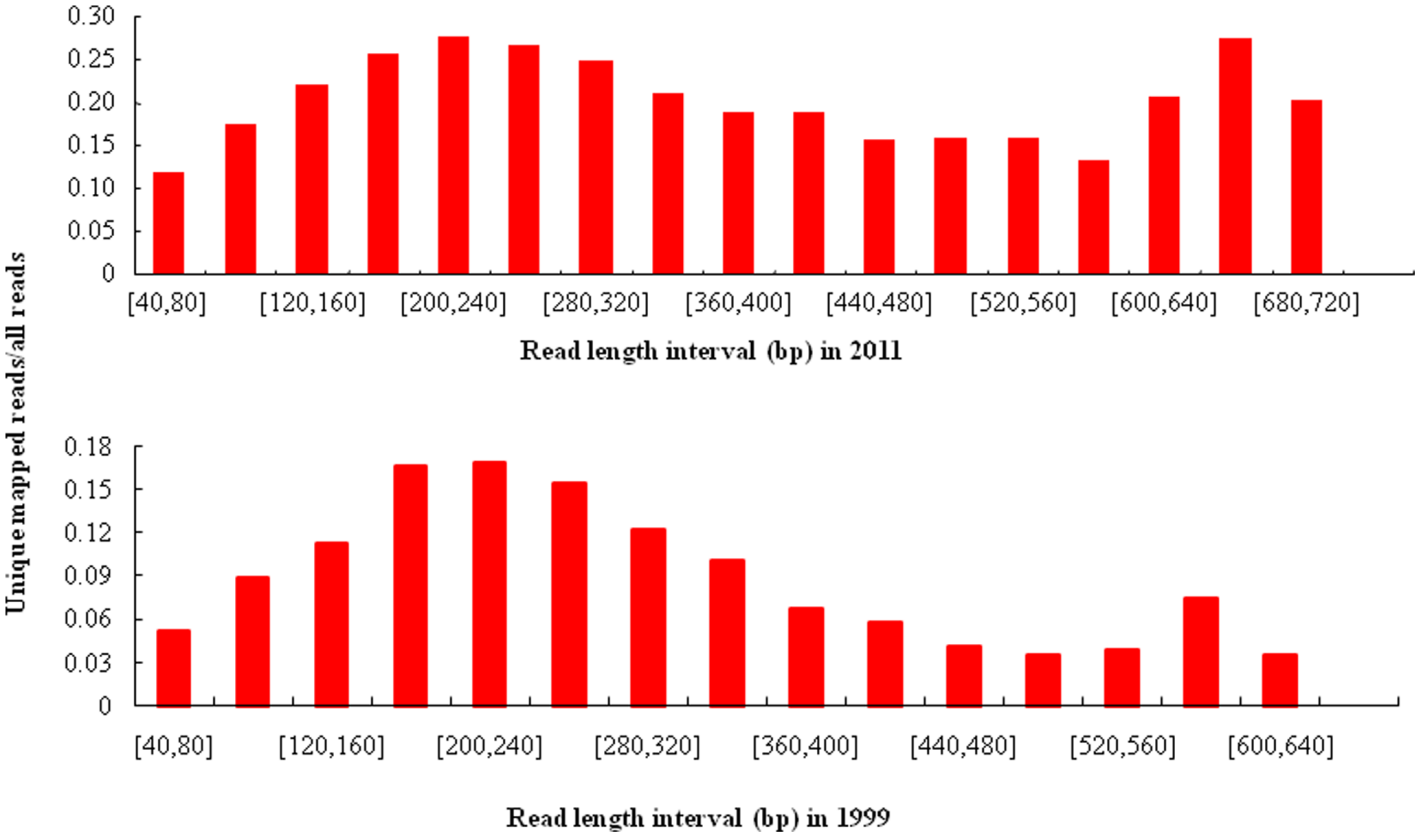
Ratio of unique mapped reads to all reads according to read length interval. A. Histogram for sample O2011. B. Histogram for sample O1999.

**Figure 6:**
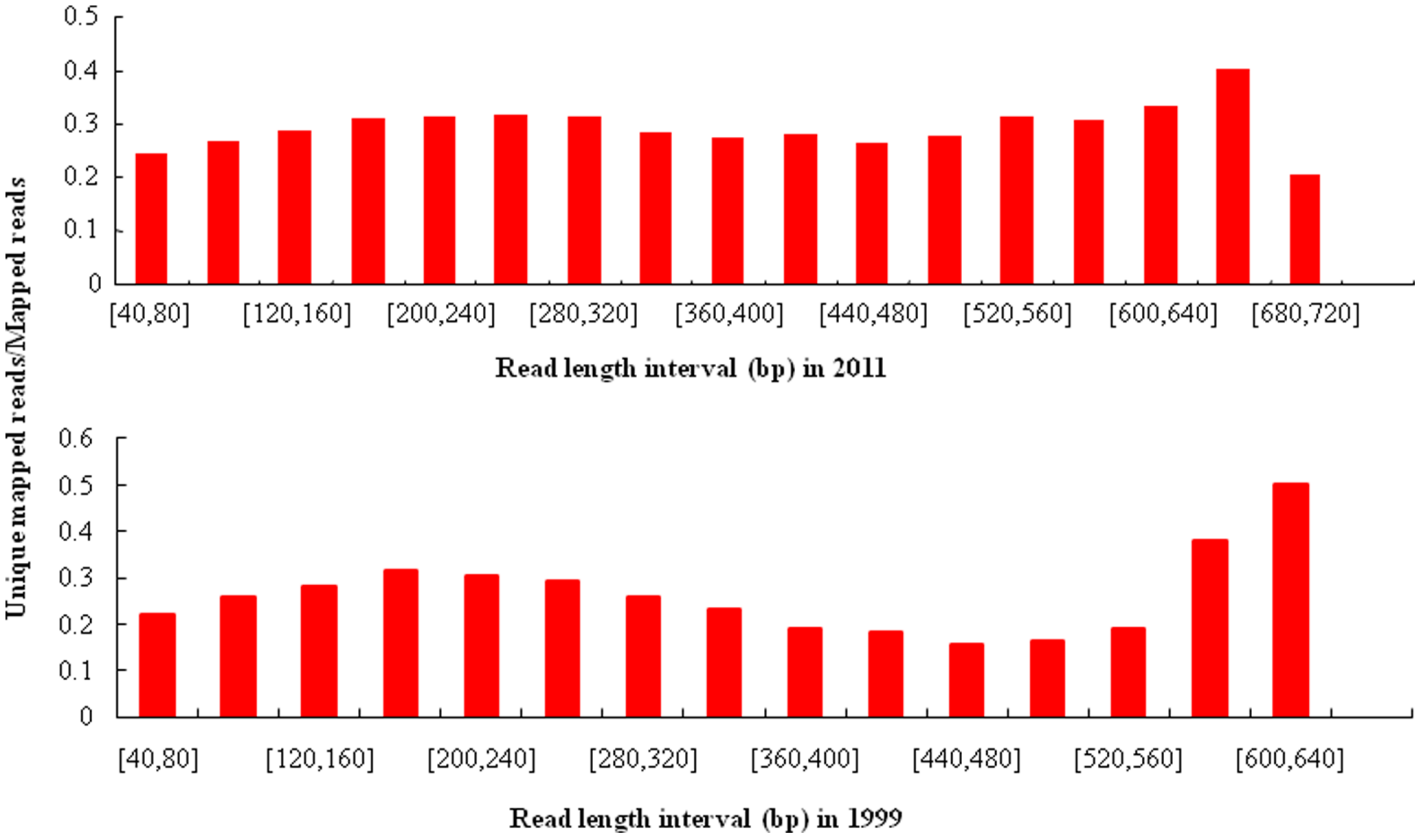
Ratio of unique mapped reads to mapped reads according to read length interval. A. Histogram for sample O2011. B. Histogram for sample O1999.

Using the bisulfite sequencing strategy described in detail in the Methods, we collected a total of 88,144 reads from the O1999 sample (data S1) and 111,527 reads from the O2011 sample (data S2) in a single run by using a fraction of a small 454 sequencing plate (25×75 Pico Titer Plate) (Tables 1–3) after filtration and quality checks. The average read lengths were 312 bp (range, 40–745 bp) and 373 bp (range, 40–657 bp) in the in O2011 and O1999 samples, respectively. After filtering out the reads mapped to short clusters of length less than 40 bp, the BWA-SW alignment program was used to align bisulfite converted sequences to the human genome (GRCh37/hg19). Bisulfite treatment efficiency was determined by calculating the C to T nucleotide conversion rate. The conversion rate for all cytosine bases was estimated to be 96.7% in O2011 and 88.6% in O1999, respectively. Of the 111,527 sequence reads in O2011, 78,738 (70.6%) were mapped to a bisulfite-converted genomic sequence (Figure 2-A) and 19.9% were uniquely mapped (Figure 3-A). The mean coverage depth was 2.5×. Of the 88,144 reads in O1999, 28,027 (31.8%) were mapped to the genomic sequence (Figure 2-B) and 7.0% were uniquely mapped (Figure 3-B). The mean coverage depth was 3.7×. The distribution of the interval lengths of the mapped reads versus all reads, and of the unique reads versus all reads, is shown in Figures 4 and 5, respectively. The distribution of the interval lengths of the unique mapped reads versus the mapped reads is shown in Figure 6. From these data, we conclude that longer sequence reads were more difficult to acquire from the O1999 tissue than from the O2011 tissue.

**Table 1:** Read length summary.

	O2011	O1999
Raw wells	257,210	175,845
Key pass wells	248,105	158,915
Passed filter wells	111,527	88,144
Total bases	34,863,012	32,961,096
Length average	312.60	373.95
Length std deviation	136.83	146.02
Longest read length	745	657
Shortest read length	40	40
Median read length	328	428

**Table 2:** Alignment summary.

	O2011	O1999
Total number of reads	111,527	88,144
Total number of reads mapped	78,738	28,027
Total number of reads mapped/Total number of reads	70.6%	31.8%
Total number of reads uniquely mapped	22,191	6,159
Total number of reads uniquely mapped/Total number of reads	19.9%	7.0%
Total length (bp) of all reads	34,863,012	32,961,096
Total length (bp) of unique mapped reads	6,719,748	1,945,099
Total length of unique mapped reads/Total length of all reads	19.3%	6.0%
Total bases of all reads	34,863,012	32,961,096
Total bases of unique mapped reads	1,181,287	285,948
Total bases of unique mapped reads/Total bases of all reads	3.4%	0.9%
Consistency rate	84.7%	83.3%
Total number of bases covered in the human genome	5.46×10^−4^	4.5×10^−5^
Depth (x-fold)	2.46	3.69

**Table 3:** Methylation summary.

Whole Sample	O2011 (%)	O1999 (%)
Conversion rate	96.7	88.6
Methylation rate of CpG on Watson	65.1	32.2
Methylation rate of CpG on Crick	42.7	45.5
Methylation rate of CpA on Watson	16.4	58.4
Methylation rate of CpA on Crick	21.7	65.8

Housekeeping Genes		
Conversion rate	90.4	74.2
Methylation rate of CpG on Watson	61.9	47.6
Methylation rate of CpG on Crick	66.9	57.5
Methylation rate of CpA on Watson	12.3	29.9
Methylation rate of CpA on Crick	40.9	48.9

### Quantitative DNA methylation analysis across the genome

The percentage of methylation at each CpG and CpA site was calculated based on the number of sequences containing unconverted cytosine (indicating methylation in the original sequence) versus the total number of sequences analyzed (Table 3). We calculated the methylation rate on both the Watson and Crick strands. Only CpG and CpA methylation rates were counted in this study because of their frequent occurrence in the human genome [8]. The methylation rates of CpG on Watson and Crick strands were 65.1% and 42.7%, respectively, in the O2011 sample, and 32.2% and 45.5%, respectively, in the O1999 sample. For CpA, the methylation rates were 58.4% and 65.8%, respectively on the Watson and Crick strands in the O1999 sample, and 16.4% and 21.7%, respectively, in the 2011 sample. We also calculated the symmetrical methylation rate and the asymmetrical methylation rate of CpG, the single methylation polymorphism rate of CpG and the single methylation polymorphism rate of CpA (Table 3).

## Discussion

With recently developed technology in the field of genetics and epigenetics, some investigators are working to refine capture protocols to reduce sample input requirements and enhance multiplexing capabilities, while others intend to refine detection to single-cell and even single-molecule resolution [2], [8]. This developing technology provides us with an opportunity to assess changes in genome-wide DNA methylation in FFPE tissues that have been stored for a long time. FFPE tissues represent the most common tissue resource used for retrospective clinical studies as well as the largest source of archival biological material. Genomic DNA isolated from archived FFPE tissue potentially has important applications, including new diagnostic assays as well as retrospective genetic and epigenetic epidemiologic studies. We also recognize the value of these samples and the potential they have to contribute to studies of the epigenome and the role of DNA methylation in numerous biological processes and diseases. Although the number of epigenetic studies continues to grow, the potential of the available FFPE samples remains largely untapped.

In this study, we evaluated methylation status by a small amount of ovarian epithelial cells obtained by Laser-directed and computer-assisted microdissection in FFPE tissue. A few studies have evaluated the use of FFPE tissue for lower throughput methylation assays that assess fewer CpG locations. Killian *et al* [9] evaluated the GoldenGate methylation assay on paired fresh and FFPE tissue from 10 lymphoma samples and 10 lymph node hyperplasia samples. They found good correlation of differentially methylated loci (DML) between fresh and FFPE samples in different groups, although the number of loci was small. Balic *et al* [7] used high-resolution melting to interrogate promoter methylation of two genes (MGMT and APC), and compared the results from paired FFPE and fresh samples in 5 human breast cancer cell lines and 3 human prostate cancer cell lines. These results were also validated with the MethyLight qPCR assay. The methylation status in archival FFPE tumor specimens from patients with colorectal cancer can be examined with high quality by high-resolution melting. Gagnon *et al* [10] validated the promoter methylation status of PLAU and TIMP3 genes in FFPE tissue by using methylation sensitive restriction enzyme digestion and qPCR. This was done for paired FFPE and fresh samples from 9 primary breast tumor samples and 4 cell line admixtures. Their data demonstrate that methylation-sensitive restriction enzyme and qPCR procedures based on digestion are good techniques for quantifying methylation percentage using limited amounts of DNA. Vilahur *et al* examined biopsies from two placentas sequentially stored at −80°C after standing at room temperature for 30 min, 1 h, 2 h, 6 h and 24 h. They found that a delay in storage at room temperature for tissue biopsies does not affect the levels of DNA methylation. Avila *et al* also found the same results for LINE-1 methylation and storage delay in the placenta using the same technique [11]. From these studies, we know that DNA methylation may not be easily degraded.

Several researchers have found that frozen tissues stored for years can yield usable nucleic acids and protein [12], [13]. One big drawback to using frozen tissue, however, is its vulnerability to thawing, and there are also financial, environmental, labor, and space issues. Other studies have evaluated the use of FFPE tissue from breast cancer cell lines, prostate cancer cell lines, primary breast tumor samples, cell line admixtures, lymphoma samples and lymph node hyperplasia samples for methylation analysis [7], [9], [10]. However, these studies involved lower throughout methylation assays, and fewer CpG locations were evaluated than in the present study. These researchers found good correlation in differentially methylated loci between fresh frozen samples and FFPE samples in different groups, in spite of the small number of loci. In a study performed using a high throughput method, it was shown that compared with frozen samples, next-generation sequenced fresh FFPE samples had smaller library insert sizes, greater coverage variability, and an increase in C to T transitions that was most pronounced at CpG dinucleotides. They concluded that there was a close relationship between DNA methylation and formalin-induced changes; but the error rate, library complexity, enrichment performance, and coverage statistics were not significantly different [14]. The most comprehensive studies involved comparisons between paired FFPE and fresh-frozen tissue samples, such as the validations reported for high-resolution melting analysis [7], qPCR quantification after bisulfite sequencing [15], methylation-specific restriction enzyme digestion [10] and Illumina's GoldenGate methylation assay [9].

Although there has been some progress in assays for DNA methylation, the recovery of DNA from FFPE tissue remains challenging. For successful long-term storage of tissue, it will be essential to understand the basis for low-level nucleic acid degradation. Causes of DNA degradation may include oxygen, humidity, light, elevated temperature and lipid peroxidation [16]. In addition, the extent of damage to the DNA may depend on the type of fixative used and on the duration of fixation [17], [18], [19], [20], [21]. Formalin fixation induces protein-protein and protein-DNA cross-linkages, and formaldehyde within the tissue gradually changes to formic acid, inducing chain breaks [17], [22], [23], [24]. In spite of these problems, it has been found that DNA extracted from FFPE tissue is fit for PCR amplification by using relatively short amplicons [25], [26]. Subsequent studies on DNA extraction from FFPE tissue have reported varying degrees of improvements such as increased amplicon length or increased effective amplifiable copy number [23], [27], [28]. Therefore, it is important to have a reliable DNA extraction method which yields DNA of high molecular weight with high quality and a low level of fragmentation. However, current DNA extraction methods do not meet the requirements for routine processing of FFPE archival materials in the clinical laboratory. The classic DNA extraction method based on long-term enzymatic digestion combined with phenol-chloroform extraction is incompatible with the development of simplified extraction protocols [23], [29], [30], [31]. The most common method to isolate genomic DNA from archival FFPE specimens is based on deparaffinization in xylene, protein digestion, followed by phenol-chloroform extraction. However, this procedure is laborious and requires the use of organic solvents, including phenols, which are carcinogens, and appropriate working environments. Many researchers have shown that fragmented DNA extracted from archival FFPE tissue rarely exceeds 300 bp, which only allows PCR analysis of short amplicons [24], [32], [33]. Several new methods have been developed and evaluated to address this challenge with varying degrees of success.

Many researchers have found that formalin fixation can cause DNA damage [9], [10], [15], [34], [35], including cross-linking, fragmentation, and generation of apurinic/apyrimidinic sites. This DNA degradation can be detrimental to qPCR [36] or whole-genome amplification [37], which are integral steps in many methylation assays. Any existing methylation assay must be carefully evaluated before it can be confidently used for FFPE-derived DNA. Promisingly, Kitazawa *et al* found that formalin fixation does not alter the methylation status of cytosine [38].

Protocols for genomic DNA extraction from FFPE specimens have been well-documented and made available as commercial kits [39], [40], but DNA extraction from FFPE tissues stored for a long time is a difficult procedure that relies on differential solubility to purify the DNA. The quality and quantity of extracted DNA and the success of subsequent DNA amplification relies on a number of parameters before, during, and after extraction. These include, but are not limited to, the amount and type of tissue, the type of fixative used for tissue preservation, the duration of fixation, the age of the paraffin block and the storage conditions, as well as the length of the desired DNA segment to be amplified [41]. Undissolved paraffin leads to poor sample quality and inhibition of PCR amplification so that removal of paraffin from the tissue is the most critical step for satisfactory extraction.

Various methods to analyze methylated DNA have been critically reviewed [18], [42], including methylation specific PCR (MSP) [43]. This technique relies on sodium bisulfite treatment of DNA, which converts unmethylated cytosine to uracil while leaving methylated cytosine unaffected. This procedure forms the basis of the commercial EZ DNA Methylation-Direct Kit. In this study, we have modified the kit manufacturer's protocol to assess DNA methylation in epithelial cells isolated by laser-directed and computer-assisted microdissection from FFPE ovarian tissues. This is the first time such a method has been used on single cells. This method is cost-effective, sensitive, and simple. Using our method, investigators now have the possibility of investigating genome-wide methylation in cells from archival FFPE tissues after many years of room temperature storage.

In this study, we chose GS Junior 454 sequencer to check reads because longer readers could be obtained (400–500 bp) [44]. In principle, the sequence reads per amplicon offers the opportunity to obtain precise quantitative methylation data for every single CpG site contained within the amplicon. The longer reads can be more accurately mapped to the reference sequence, especially reads from methylated sequences, which contain only three different bases. The 454/Roche platform can reliably detect and quantify the degree of methylation of partially methylated reads compared with the other platform. However, the 454/Roche platform generates fewer reads. The max throughout of this sequencer is only 30 Mb. Owing to the low coverage, the coverage rates in the human genome are only 5.46×10^−4^ and 4.5×10^−5^ in 2 samples. Therefore, the methylation status of a specific locus may not always be reliably determined. We may resolve this problem by multiple measurements.

One limitation of this study is that those two samples used in this study were from two different individuals. However, we believe data from the same individual with different length of storage would be more valuable, because this design may eliminate potential confounding by unmeasured or unknown factors related to the study participants. The other limitation is the small sample size (n = 2), which does not allow any statistical test. We anticipate that a large study will be carried out in the future.

## Methods

### Patient samples

FFPE blocks containing normal ovarian tissue samples that had been surgically removed from two older patients because of uterine leiomyoma were used for histopathology and methylation analysis. These FFPE tissue blocks had been stored in the archives of the Pathology Department of the First Affiliated Hospital of Xi'an Jiaotong University for about 13 years (O1999) and less than 1 year (O2011) at the time of analysis. Histopathology was performed independently by two histopathologists, and there was concordance between them. The clinical characteristics of two patients are listed in Table 4. This study was approved by the Institutional Review Board (IRB) in Xi'an Jiaotong University, and written informed consent was obtained from both patients.

**Table 4:** The clinical characteristics of two patients.

Age/menstruation	History of reproduction	Ultrasound examination	Diagnosis*	Date of operation**
52 y.o./postmenopause	2-1-1-2	an enlarged uterus measuring 12.6×8.9×9.8 cm with normal-size ovaries	Uterine leiomyomas	August 10, 1999
54 y.o./postmenopause	3-0-0-3	an enlarged uterus measuring 14.3×10.4×8.6 cm with normal-size ovaries	uterine leiomyomas	April 23, 2010

*Pathological study: uterine leiomyomas with normal ovaries.

**Operation: Total hysterectomy and bilateral salpingo-oophorectomy.

### H&E staining

Ten-micrometer tissue sections were used for all subsequent analyses. Sections were mounted on membrane-covered slides (Leica Microsystems; Wetzlar, Germany). The paraffin was removed prior to staining the paraffin-embedded sections by washing the slides with xylene followed by a series of descending concentrations of ethanol as follows: 3× xylene 20 seconds (three separate containers), 2×100% ethanol 30 seconds (two separate containers), 2× 95% ethanol 30 seconds, 2×70% ethanol 30 seconds, distilled H_2_O 30 seconds. Staining was performed as follows: hematoxylin 10 seconds, H_2_O 1 min, eosin solution 10 seconds, distilled H_2_O 1 min, 70% ethanol 30 seconds, 95% ethanol 30 seconds, air-dry at room temperature.

### Epithelial cells acquired by laser microdissection

**Figure 7:**
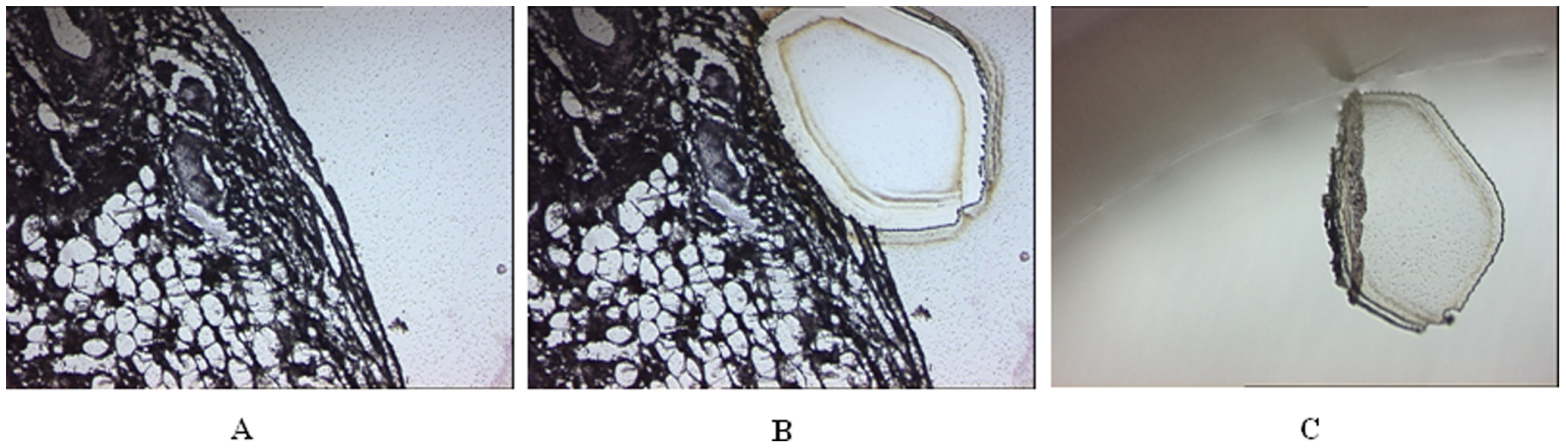
The process of micro-dissection using the Leica LMD 7000. Ovarian epithelial cells in a normal ovarian tissue section before micro-dissection (A), the same section after cell selection and micro-dissection (B), and the micro-dissected cells in water (C). Original magnification, x10.

The microdissection of ovarian epithelial cells from normal human ovarian tissue was performed using a laser-directed and computer-assisted microdissection microscope (Leica AS LMD 7000; Leica Microsystems) (Figure 7, A–C) with a pulsed 337 nm UV laser. Captured single cells were dissected out and collected into individual tubes (EU single thin-wall 0.2 ml tube with cap, BIO plastics) filled with 20 µl DNase/RNase-free water (Invitrogen, 75-0024). The tubes were centrifuged at full speed (>10,000×g) for 5 minutes, after which the top 11 µl of water was discarded. All experiments were carried out in duplicate.

### Genomic DNA bisulfite conversion

The EZ DNA Methylation-Direct Kit (ZYMO RESEARCH, D5021) was used to bisulfite-convert DNA from single cells. Ten µl of M-Digestion Buffer and 1 µl of Proteinase K were added to 9 µl of sample liquid for digestion. Each sample was incubated for 4 h at 50°C. About 20 µl of sample was added to 130 µl of CT Conversion Reagent solution in a PCR tube. Samples were mixed and then centrifuged briefly to ensure that no droplets were in the cap or on the sides of the tube. The PCR tubes were placed in a thermal cycler and the following conditions were used: 98°C for 8 minutes, 64°C for 7 h (we increased the conversion time from 3.5 h in the manufacturer's protocol to 7 h in order to obtain better conversion rate), 4°C storage for up to 20 h. About 600 µl of M-Binding Buffer was added to each Zymo-Spin IC Column, and the prepared columns placed into the provided collection tubes. The DNA samples were loaded into the prepared columns, after which the caps were closed, the samples mixed by inverting the column several times, and the tubes centrifuged at full speed (>10,000×g) for 30 seconds. The flow-through was discarded. About 200 µl of M-Desulphonation Buffer was added to each column. The columns were allowed to incubate at room temperature (20–30°C) for 15–20 minutes, and then centrifuged at full speed for 30 seconds. About 200 µl of M-Wash Buffer was added to each column, and then the columns were centrifuged at full speed for 30 seconds. Another 200 µl of M-Wash Buffer was added and the centrifugation step repeated for an additional 30 seconds. Each column was then placed into a 1.5 ml micro centrifuge tube. About 10 µl of M-Elution Buffer was added directly into the column matrix, and the columns were centrifuged for 30 seconds at full speed to elute the DNA.

### Whole genome amplification (WGA) of bisulfite converted DNA

Whole genomic amplification of bisulfite converted DNA was performed using the EpiTect Whole Bisulfitome Kit (QIAGEN, 1052668). Bisulfite converted template DNA was placed into a micro centrifuge tube. The volume was adjusted to 10 µl using nuclease-free water. REPLI-g Midi DNA polymerase was thawed on ice. All other components were thawed at room temperature, vortexed, and then centrifuged briefly. An EpiTect Amplification Master Mix was prepared on ice, containing 29 µl of EpiTect WBA Reaction Buffer and 1 µl of REPLI-g Midi DNA polymerase. 30 µl of EpiTect Amplification Master Mix was added to 10 µl of bisulfite converted DNA. The solution was incubated at 28°C for 8 h. REPLI-g Midi DNA Polymerase was inactivated by heating the sample for 5 min at 95°C. The amplified DNA was stored at 4°C for short-term storage or −20°C for long-term storage.

### Purification of amplified bisulfite converted genomic DNA

Purification of PCR products was performed using the QIAquick PCR purification kit (QIAGEN, 28106). For each sample, 5 volumes of Buffer PB were added to 1 volume of the PCR product and mixed. A QIA spin column was placed into a 2 ml of collection tube. To bind DNA, the sample was applied to the QIAquick column and centrifuged for 30–60 seconds. The flow-through was discarded, and the QIAquick column was placed back into the same tube. 0.75 ml of Buffer PE was added to the QIAquick column, which was then centrifuged for 30–60 seconds to wash the DNA. The flow-through was discarded and the QIAquick column was placed back in the same tube. The column was centrifuged for an additional 1 min at maximum speed. The QIAquick column was placed in a clean 1.5 ml microcentrifuge tube. To elute the DNA, 50 µl of H_2_O was added to the center of the QIAquick membrane and the column was centrifuged for 1 min. All DNA concentrations were measured by Nanodrop (Thermo-Fisher, USA).

### Sequencing using the GS Junior 454 sequencing system

The purified PCR products of the bisulfite-treated DNA fragments were end-repaired and ligated to sequencing adaptors using 454 library construction kits, and sequenced according to the manufacturer's protocols (454 Life Sciences, a subsidiary of Roche, Branford, CT). Emulsion PCR and sequencing were performed according to the standard protocols from the 454 system.

### Sequencing data analysis

Statistical analyses were conducted using computer programs. The sequence reads were mapped to the human genome (GRCh37/hg19) using the mapping program BWA-SW (http://bio-bwa.sourceforge.net) [19]. The mapping process was divided into two phases to model the possible bisulfite conversions, i.e., C to T and G to A. For each phase, Cs (Gs) to Ts (As) were converted in both the reads and the reference sequences. To each read was added a special character, ‘a’ or ‘b’, to distinguish its conversion (a: C to T and b: G to A). Based on the converted reads and reference sequences, BWA was implemented as following: Step 1: Mapping reads. The two versions of reads were both mapped to the Watson strand and the Crick strand. Here, the parameter Z was set as 50 and all other parameters set to their default values. Step 2: Filtering repeats. For every mapped read, the mapped location was tagged as ‘M’ in the mapping result. RepeatMasker, Segmental Dups and DGV Struct Var were used to identify repeats in reads. If a read included the following types of repeats: Alu, simple repeats, ERVL-MaLR, low-complexity, L1HS, L1M, L1P, LTR ERV, Segmental Dups and CNVs, the repeat locations were marked in the read. The read was removed if it satisfied either of the following two cases: 1) it included two different types of repeats; or 2) the unmarked ‘M’ length in it was less than 20 bp. Step 3: Filtering overlaps. A read may have multiple mapping results. Only the one that had the shortest overlap with RepeatMasker/CNV/segmental dups was kept. For two reads converted from the same read, if one read's mapping segments were covered totally by the other read, the covered one was removed. For a given read, if its mapping segments were partly overlapped by another read and the non-overlapped ‘M’ segments were not more than 20 bp, it was removed. Step 4: Repeated mapping. For any unmapped reads longer than 35 bp, they were separated and steps 1–3 were repeated.

The bisulfite conversion rate was calculated as the number of genomic cytosines outside a CpG context that were unconverted, divided by the total number of cytosines outside a CpG context. To calculate the methylation index, the methylation status of each CpG site in each sequence read was first determined based on a C to T conversion at each CpG site on the forward strand and a G to A conversion on the reverse strand. The percentage of methylation at each CpG site was calculated based on the number of sequences containing methylated CpG sites versus the total number of sequences analyzed. If multiple reads mapped on the same CpG position either on the Watson strand or on the crick strand, then we can calculate the single-methylation polymorphism. That is, the reads number that are mapped to same CpG of Watson (or Crick) strands, some reads include CG but another reads include TG versus all reads number that are mapped to same CpG of Watson (or Crick) strands.

A list of housekeeping genes were downloaded from the database http://www.tau.ac.il/~elieis/HKG/, in combination with the housekeeping genes reported by Eisenberg and Levanon 2013 and Butte et al, 2001 [45], [46]. The data on these housekeeping genes were extracted and summarized in Table 3.

## Supporting Information

Data S1 **The original Roche 454 data of sample O1999.**
(ZIP)

Data S2 **The original Roche 454 data of sample O2011.**
(ZIP)
